# Impact of opioid abuse on oral health: a retrospective cohort study

**DOI:** 10.3389/froh.2025.1483406

**Published:** 2025-02-21

**Authors:** Martyna Smeda, Constanze Knogl, Karolina Müller, Martin Stahl, Wolfgang Buchalla, Lukas Keim, Ursula Piendl, Norbert Wodarz, Matthias Widbiller

**Affiliations:** ^1^ Department of Conservative Dentistry and Periodontology, University Hospital Regensburg, Regensburg, Germany; ^2^ Center for Clinical Studies, University Hospital Regensburg, Regensburg, Germany; ^3^ Center of Addiction Medicine of the Department of Psychiatry and Psychotherapy, University of Regensburg, Regensburg, Germany

**Keywords:** opioid, heroin, addiction, oral health, oral hygiene, periodontal diseases, saliva

## Abstract

**Objectives:**

Opioid use has significantly increased in Germany in recent years. This study aimed to evaluate and compare the oral health, dental hygiene, self-perceived pain, and functional limitations of opioid-addicted patients with a healthy control group.

**Materials and methods:**

50 opioid-addicted patients (OAP) attending substitution treatment at the Centre for Addiction Medicine of the Department of Psychiatry and Psychotherapy at the University of Regensburg were enrolled and interviewed about their drug use history. A control group was matched for age and sex. The oral health status of patients in both groups was documented using a record of decayed, missing and filled teeth (DMFT), Periodontal Screening Index (PSI), Silness and Loe Plaque Index (PI), stimulated salivary flow rate, buffer capacity and pH. Patients also completed a questionnaire on oral hygiene, functional limitations, dietary habits and other topics. Statistical analysis was performed using non-parametric tests (*α* = 0.05).

**Results:**

More men (74%) than women (26%) participated in the study with an age range of 19–64 years. According to the inclusion criteria, all OAP had a history of heroin use, followed by cannabinoids (90%) and cocaine (82%). The median DMFT of the OAP was 21 (IQR = 12–28) and was significantly higher (*P* < 0.001) than in the control group (median = 10, IQR = 5–16). In addition, the OAP had a significantly higher proportion of periodontal treatment needs (*P* < 0.001). The PI of the OAP was also significantly worse (*P* = 0.012). The pH of stimulated saliva from the OAP (median = 6.8, IQR = 6.4–7.2) was significantly lower (*P* = 0.002) compared to the control group (median = 7.2, IQR = 7.0–7.4). However, there were no significant differences in stimulated salivary flow rate and buffering capacity (*P* > 0.086). OAP had significantly poorer oral hygiene, with a particular lack of interdental care (*P* ≤ 0.0012), and a significantly higher consumption of sweets or sweetened drinks appeared to be a problem (*P* ≤ 0.027). Functional limitations (*P* < 0.001) were reported to be a burden for OAP.

**Conclusion:**

Opioid addiction significantly impacts oral health, necessitating improved dental care and confidential treatment services to prevent dental and periodontal diseases and support the social integration of affected individuals.

## 1 Introduction

Opioid addiction is multifaceted with significant health implications for individuals (1, 2). Opioids, which include naturally occurring, semi-synthetic and synthetic substances, bind to opioid receptors in the brain and produce sedative, analgesic and euphoric effects (3, 4). These drugs are commonly used to treat severe pain, including cancer-related pain (5). The US Drug Enforcement Association (DEA) classifies opioids according to their medical use, potential for abuse and risk of dependence. Well-known opioids include medically used substances such as morphine and codeine, which vary in their addictive potential, and non-medically used drugs such as heroin (6).

Opioid abuse is both a psychological and a physical condition (1, 2). It can result from a number of factors, including genetic predisposition, stress, prolonged use of opioid analgesics, and an individual's circumstances and environment. These factors, combined with the increasing availability of illicit opioids, have contributed to the opioid epidemic, particularly in the US (1, 7).

Since the 1990s, the development of more opioid-based drugs by pharmaceutical companies, together with effective advertising campaigns and low costs, has led to a significant increase in prescription rates. Patient privacy laws and a lack of communication across state borders prevented the monitoring of prescription quantities per patient, facilitating the sale of excess opioids on the black market (8–14). The increasing availability of opioids and their high potential for addiction led to a sixfold increase in opioid-related deaths in the US between 1999 and 2017, surpassing HIV-related deaths during the AIDS epidemic (12–15). By November 2017, the annual cost of the opioid crisis in the US had reached $ 504 billion, underscoring its status as a national problem (13, 16).

Similarly, opioid use and prescribing have increased substantially in many European countries since 2004, although not to the same extent as in the US (17). According to a report of the International Narcotics Control Board in 2022, Western and Central Europe ranked second in the world in terms of “defined daily doses (S-DDD) of opioid analgesics per million inhabitants per day” at around 8,500 in 2021 (18). Almost all European countries have experienced an upward trend in prescribed opioid use since 2008, with Germany and Austria showing particularly high rates (19, 20). Projections from addiction treatment centers indicate that the number of opioid addicts in Germany was 166,294 in 2016, suggesting a consistent trend. The rate of opioid addiction varies between the German federal states, with Bavaria's rate estimated at 1.3 per 1,000 inhabitants, placing it in the middle range of the federal states (21).

Despite the well-known effects of opioid addiction on general health, such as constipation, cognitive problems and sleep disorders, oral health is often neglected (22). Drug abuse significantly alters behaviors and lifestyle, leading patients to de-prioritize dental health. Poor oral hygiene can lead to caries, periodontal disease and tooth loss. In addition, changes in self-evaluation and self-perception can lead to carelessness and poor hygiene, making patients less likely to seek dental care (23–25). To date, no study has investigated the oral health of opioid dependent patients in comparison with healthy patients in a European country (25).

Therefore, the primary objective of this retrospective cohort study was to evaluate oral health and dental hygiene of patients with opioid addiction compared with a healthy control group. Specifically, the study examined several aspects including dental status, periodontal status, salivary parameters, dental care and dietary habits. In addition, the study sought to assess patients’ self-perceived pain and functional limitations. The null hypothesis was that these factors would not differ significantly between opioid-addicted patients (OAP) and non-opioid-addicted patients (non-OAP).

## 2 Material and methods

### 2.1 Patient recruitment

For this retrospective cohort study, 50 opioid-addicted patients undergoing substitution treatment at the Centre for Addiction Medicine of the Department of Psychiatry and Psychotherapy at the University of Regensburg were enrolled after obtaining informed consent as approved by the Ethics Committee of the University of Regensburg (Number: 20-2031-101). The OAP cohort was compared with a control group of patients with no history of drug use, matched for sex and age. The control group was recruited from the Department of Conservative Dentistry and Periodontology at the University Hospital Regensburg.

### 2.2 Drug history and substitution

Before the clinical examination, the participants were questioned in detail about their history of drug abuse. The age at which they first used an addictive substance, the age at which they became regular users and the total duration of use were recorded. It was also noted if the participant had taken the substance within four weeks of the examination. In addition, it was documented which substitutes were administered, in what form and at what dose.

### 2.3 Oral health status

The dental examination was carried out equally in the test and control groups. Decayed (D), missing (M) and filled (F) teeth (T) were recorded and the DMFT index was calculated. The Periodontal Screening Index (PSI) was obtained to determine the need for periodontal treatment (26) and the Plaque Index (PI) by Silness and Loe to assess oral hygiene (27). In addition, stimulated salivary flow rate, pH and buffering capacity were measured using the GC Saliva-Check Buffer (GC Corporation, Tokyo, Japan).

### 2.4 Oral health questionnaire

Participants in both groups were asked to complete a questionnaire with single- and multiple-choice questions on demographics, oral hygiene habits and dietary behaviors. There were also questions on self-perception of dental status and oral health-related quality of life, such as experienced discomfort or pain due to oral problems. The source for the standardized questions was the WHO manual for oral health surveys (28). The survey was completed independently by the participants without any assistance by the investigators.

### 2.5 Statistical analysis

Statistical analysis was performed using IBM SPSS Statistics for Windows, version 29.0 (IBM Corp., Armonk, NY, USA). Most data were not normally distributed and therefore non-parametric tests were conducted at a significance level of *α* = 0.05. Mann–Whitney *U* tests or Chi-squared tests were applied depending on the scale of measurement.

## 3 Results

### 3.1 Study population

Overall, more men (74%) than women (26%) were included in the study (Table 1). The mean age of the participants was 39 years (SD = 10), ranging from 19 to 64 years. Most opioid-addicted patients grew up in a middle-class household (74%), as did most patients in the control group (90%). There were no significant differences between OAP and non-OAP regarding their social background (*P* = 0.317). However, there were statistically significant differences in place of residence (*P* < 0.001) and education (*P* < 0.001). The majority of OAP reported living in the city (68%), while many non-OAP reported residing in the country (46%). Most of the OAP had a lower level of education than the control group. 14% of the OAP but none of the control group had not finished school.

**Table 1:** Study population characteristics. Data are expressed as total numbers *n* (%). Abbreviations: opioid addicted patients (OAP), non-opioid addicted patients (non-OAP).

	OAP	non-OAP	*P*-value
Total	50 (100)	50 (100)	
Male	37 (74)	37 (74)	
Female	13 (26)	13 (26)	
Age			
Mean ± STD [Min–Max]	39 ± 10 [19–64]	39 ± 10 [19–64]	
Median, IQR	39, 31−44	38, 31–44	
Social status growing up (survey question 14)			
Lower class	9 (18)	3 (6)	0.317
Middle class	37 (74)	45 (90)	
Upper class	4 (8)	2 (4)	
Living environment (survey question 12)			
City	34 (68)	20 (40)	<0.001
Suburbs	10 (20)	7 (14)	
Country	6 (12)	23 (46)	
Completed school career (survey question 13)			
None	7 (14)	0 (0)	<0.001
Special school	0 (0)	0 (0)	
Comprehensive school	29 (58)	15 (30)	
Secondary school	10 (20)	11 (22)	
Academic high school	4 (8)	24 (48)	

### 3.2 Drug history and substitution

As shown in Table 2, heroin was the most common opioid used by all OAP, followed by cannabinoids (90%), cocaine (82%) and amphetamines (80%). In early adolescence, the most common gateway drugs among surveyed drug users were sniffing substances [median age at first use: 14 years (IQR = 12–16)], alcohol [median age at first use: 14 years (IQR = 13–16)] and cannabinoids [median age at first use: 14 years (IQR = 13–16)]. In late adolescence and young adulthood, opioids such as amphetamines [median age at first use: 17 years (IQR = 15–20)], heroin [median age at first use: 18 years (IQR = 16–20)] and cocaine [median age at first use: 18 years (IQR = 16–23)] were consumed. With a median total duration of use of 22 years [IQR = 12–28], alcohol is the most common constant companion of opioid users, followed by cannabinoids [total duration of consumption: 18 years (IQR = 11–27)].

**Table 2:** Details of substances/drugs used in the group of opioid-addicted patients (OAP), sorted in ascending order by age at first use. Data are expressed as Median, IQR [Min–Max] or *n* (%).

	Participants	Age at first use	Age with regular use	Years of consumption	Use in the past month
Sniffing substances	5 (10)	14, 12–16 [10–16]	13^a^ *(4 missing)*	3^a^ [1–5] *(3 missing)*	0 (0)
Alcohol (any use)	28 (56)	14, 13–16 [8–30]	15, 14–16 [13–30] *(17 missing)*	22, 12–28 [3–37] *(5 missing)*	8 (29)
Cannabinoids	45 (90)	14, 13–16 [11–20]	15, 13–16 [11–36] *(11 missing)*	18, 11–27 [2–48] *(5 missing)*	18 (40)
Alcohol (to drunkenness)	25 (50)	16, 14–30 [11–44]	16, 14–29 [11–49] *(2 missing)*	15, 6–22 [2–31] *(2 missing)*	13 (52)
Amphetamines	40 (80)	17, 15–20 [12–40]	16, 15–19 [12–22] *(15 missing)*	14, 8–21 [0.5–34] *(6 missing)*	12 (30)
Heroin	50 (100)	18, 16–20 [13–40]	18, 17–22 [14–40] *(4 missing)*	14, 8–24 [1–44]	25 (50)
Hallucinogens	28 (56)	18, 16–22 [12–37]	23, 17–25 [12–25] *(22 missing)*	5, 2–15 [0.1–23] *(8 missing)*	0 (0)
Cocaine	41 (82)	18, 16–23 [12–45]	17, 15–22 [12–39] *(27 missing)*	15, 5–21 [1–34] *(7 missing)*	7 (17)
Tablets^b^	37 (74)	19, 16–25 [13–41]	20, 17–26 [13–30] *(17 missing)*	14, 8–24 [1–30] *(3 missing)*	22 (60)
Other opiates or analgesics	29 (58)	20, 17–28 [14–40]	21, 18–29 [15–37] *(15 missing)*	10, 4–16 [0.5–26] *(6 missing)*	7 (24)
Other substances	4 (8)	22, 14–30 [14–30]	29^a^ [15–30] *(1 missing)*	11^a^ [3–19] *(1 missing)*	1 (25)
Substitution medication^c^	50 (100)	25, 20–34 [15–60]	25, 21–35 [17–60] *(10 missing)*	10, 5–15 [1–26] *(5 missing)*	46 (92)

^a^ Only medians given due to low number of valid values.

^b^ Benzodiazepines, barbiturates, sedatives, hypnotics, tranquillizers.

^c^ Methadone, buprenorphine.

Participants in this study received substitution treatment for the first time at a median age of 25 years [IQR = 20–34] (Table 2). However, many of them reported at the time of the survey that they had continued to use opioids in the four weeks prior to the survey. The majority of OAP (62%) were receiving L-Polamidon as a substitution medication, with liquid (84%) being the most common form of administration (Table 3). 18% of the test group received Subutex tablets, 12% Methaddict tablets, 4% Buprenaddict tablets, 2% Buprenorphin in form of a sublingual or buccal film and 2% L-Polaflux liquid. Two of the patients were given a second substitution substance as described in Table 3.

**Table 3:** Substitution medication and form administered to opioid-addicted patients (OAP). Quantity expressed as total numbers *n* (%), and individual pharmaceutical forms (*n*) including dosages (Mean ± STD) are presented.

	Participants	Juice	Tablet	Solution	Subl. tablet	Powder
Polamidon	31 (62)	26 (31 ± 26 mg)	4 (45 ± 21 mg)	1 (9.6 mg)		
Subutex	9 (18)		7 (11 ± 3 mg)		2 (6 ± 3 mg)	
Methaddict	6 (12)		6 (68 ± 40 mg)			
Buprenaddict	2 (4)		2 (12 mg)			
Buprenorphin	1 (2)					1 (4 mg)
Polaflux	1 (2)	1 (40 mg)				

### 3.3 Oral health status

The DMFT, a measure of dental health, was significantly higher in OAP with a median of 21 (IQR = 12–28) compared to non-OAP with a median DMFT of 10 (IQR = 5–16) (*P* < 0.001) (Table 4). OAP were found to have significantly more decayed and missing teeth (*P* < 0.001). In addition, OAP had significantly fewer filled teeth (*P* < 0.001) than patients in the control group.

**Table 4:** Decayed, missing or filled teeth (DMFT). Median, IQR [Min–Max] of opioid addicted patients (OAP) and non-opioid addicted patients (non-OAP).

	OAP	non-OAP	*P*-value
DMFT	21, 12–28 [1–28]	10, 5–16 [0–23]	<0.001
D (decayed)	5, 2–9 [0–22]	0, 0–0 [0–4]	<0.001
M (missing)	5, 1–13 [0–28]	0, 0–1 [0–13]	<0.001
F (filled)	4, 1–7 [0–22]	8, 5–13 [0–21]	<0.001

As shown in Table 5, periodontal status assessed by PSI showed a significantly higher need for periodontal treatment, represented by grades 3 and 4, in the OAP (*P* < 0.001).

**Table 5:** Existence or severity of periodontal disease by the highest value of the periodontal screening index (PSI) of each patient. Data are expressed as *n* (%). Abbreviations: opioid addicted patients (OAP), non-opioid addicted patients (non-OAP).

	OAP^a^	non-OAP	*P*-value
No periodontitis (PSI grade 0, 1 or 2)	9 (19)	35 (70)	<0.001
Periodontitis (PSI grade 3 or 4)	38 (81)	15 (30)	

^a^ Three edentulous patients in the OAP group and therefore only 47 valid values.

According to the PI, 29.8% of opioid users had poor oral hygiene (Table 6). The PI of OAP was significantly higher than that of non-OAP (*P* = 0.012), indicating worse oral hygiene in OAP.

**Table 6:** Assessment of dental hygiene using the plaque index after silness and Loe (PI). Data are expressed as *n* (%). Abbreviations: opioid addicted patients (OAP), non-opioid addicted patients (non-OAP).

		OAP^a^	non-OAP	*P*-value
PI	Very good	6 (12.8)	7 (14)	0.012
	Good	16 (34)	29 (58)	
	Satisfactory	11 (23.4)	11 (22)	
	Poor	14 (29.8)	3 (6)	

^a^ Three edentulous patients in the OAP group and therefore only 47 valid values.

Regarding the saliva parameters, no statistically significant differences were found between OAP and non-OAP for salivary flow rate (*P* = 0.266) or buffering capacity (*P* = 0.086) (Table 7). However, a significant difference was found for salivary pH. It was significantly more acidic (*P* < 0.001) with a median pH of 6.8 (IQR = 6.4–7.2) in the OAP group compared to the control group with a median pH of 7.2 (IQR = 7.0–7.4).

**Table 7:** Stimulated saliva flow rate, pH and buffer capacity. Data are expressed as Median, IQR [Min–Max] or *n* (%). Abbreviations: opioid addicted patients (OAP), non-opioid addicted patients (non-OAP).

		OAP	non-OAP	*P*-value
Flow rate in ml/min		1.2, 0.95–1.65 [0.2–3.4]	1.4, 1.15–1.6 [0.4–3.0]	0.266
pH		6.8, 6.4–7.2 [5.8–7.6]	7.2, 7.0–7.4 [6.6–7.6]	0.002
pH—valuation	Very acidic	2 (4)	0 (0)	<0.001
	Acidic	19 (38)	6 (12)	
	Healthy	29 (58)	44 (88)	
Buffer capacity	Very low	16 (32)	2 (4)	0.086
	Low	12 (24)	24 (48)	
	Normal-high	22 (44)	24 (48)	

### 3.4 Oral health questionnaire

Table 8 shows that OAP were significantly more likely to describe the condition of their teeth and gums as poor or very poor (*P* < 0.001). 58% of the opioid users surveyed considered that they had fewer than 20 teeth. Among patients in the control group, this proportion was only 8% resulting in statistically significant differences in the self-assessment of the number of teeth (*P* < 0.001). Overall, 34% of OAP reported having removable dentures. They were significantly more likely to report having a complete denture in the upper jaw (*P* = 0.001) or lower jaw (*P* = 0.027).

**Table 8:** Results of the oral health questionnaire. Data are expressed as *n* (%). Abbreviations: opioid addicted patients (OAP), non-opioid addicted patients (non-OAP).

	Answer	OAP	non-OAP	*P*-value
How many natural/own teeth do you have?	None	7 (14)	0 (0)	<0.001
	1–9	7 (14)	0 (0)	
	10–19	15 (30)	4 (8)	
	20 or more	21 (42)	46 (92)	
Do you have removable dentures and if so, which ones?^a^	Partial denture	1 (2)	1 (2)	1.0
	Full denture upper jaw	10 (20)	0 (0)	0.001
	Full denture lower jaw	6 (12)	0 (0)	0.027
	I do not have removable dentures	38 (76)	49 (98)	0.002
How would you describe the condition of your teeth and gums?	Excellent	0 (0)	2 (4)	<0.001
	Very good	1 (2)	7 (14)	
	Good	7 (14)	17 (34)	
	Average	15 (30)	20 (40)	
	Poor	13 (26)	3 (6)	
	Very poor	14 (28)	1 (2)	
	I can't judge it	0 (0)	0 (0)	
How often do you clean your teeth?	Never	2 (4)	0 (0)	<0.001
	Once per month	0 (0)	0 (0)	
	2–3 times per month	2 (4)	0 (0)	
	Once per week	1 (2)	0 (0)	
	2–6 times per week	3 (6)	2 (4)	
	Once a day	27 (54)	14 (28)	
	2 times or more per day	15 (30)	34 (68)	
Which of the following products do you use to clean your teeth?^a^	Toothbrush	48 (96)	48 (96)	1.0
	Mouthwash	27 (54)	27 (54)	1.0
	Chewing gum	8 (16)	7 (14)	1.0
	Dental floss	7 (14)	31 (62)	<0.001
	Wooden toothpick	4 (8)	7 (14)	0.525
	Plastic toothpick	4 (8)	1 (2)	0.362
	Interdental brushes	3 (6)	13 (26)	0.012
	Others	1 (2)	3 (6)	0.617
Do you use toothpaste to brush your teeth?	Yes	48 (96)	49 (98)	1.0
	No	2 (4)	1 (2)	
Does your toothpaste contain fluoride?	Yes	23 (46)	35 (70)	0.039
	No	4 (8)	1 (2)	
	I do not know	23 (46)	14 (28)	
How long ago was your last visit to the dentist?	Less than 6 months	19 (38)	24 (48)	0.027
	6–12 months	12 (24)	20 (40)	
	Between 1 and 2 years	9 (18)	5 (10)	
	Between 2 and 5 years	4 (8)	1 (2)	
	5 or more years	6 (12)	0 (0)	
	I've never been to the dentist	0 (0)	0 (0)	
What was the reason for your last visit to the dentist?	Pain or other discomfort	28 (56)	8 (16)	<0.001
	Treatment/Aftercare	16 (32)	9 (18)	0.165
	Counselling	5 (10)	2 (4)	0.436
	Routine check	4 (8)	33 (66)	<0.001
	I don't know/can't remember	3 (6)	0 (0)	0.242
Have you had any pain or other discomfort in your mouth in the last 12 months?	Yes	32 (64)	12 (24)	<0.001
	No	17 (34)	37 (74)	
	I don't know	1 (2)	1 (2)	

^a^ Multiple answers were possible.

Statistically more OAP reported brushing their teeth less than once a day (*P* < 0.001). Only 30% of the drug users reported brushing their teeth twice or more a day, compared with 68% of controls. There were no significant differences between OAP and non-OAP in the use of toothbrushes (*P* = 1.0), mouthwashes (*P* = 1.0), chewing gum (*P* = 1.0), wooden toothpicks (*P* = 0.525) or plastic toothpicks (*P* = 0.362). However, opioid users were significantly less likely to use dental floss (*P* < 0.001) or interdental brushes (*P* = 0.012). The majority of patients in the test group (96%) and control group (98%) used toothpaste to brush their teeth, but OAP were significantly less likely to know whether their toothpaste contained fluoride (*P* = 0.039).

The questionnaire revealed that OAP were less likely to visit the dentist (*P* = 0.027). 12% reported that it had been 5 years or more since their last visit to the dentist. The reason for visiting a dentist was significantly more often due to pain or other complaints (*P* < 0.001). OAP were shown to be significantly less likely than non-OAP to attend routine check-ups (*P* < 0.001). In addition, 64% of the opioid-using respondents reported having experienced pain or other complaints in the oral cavity in the past 12 months, which was significantly different from the control group (*P* < 0.001).

### 3.5 Oral health-related quality of life questionnaire

The questions on dental and the masticatory function showed that OAP are more likely to have complaints that lead to a reduced quality of life (Table 9). Opioid users were significantly more likely to have problems with biting, chewing and dry mouth (*P* < 0.001). They often reported feeling embarrassed in public because of the condition of their teeth and even avoided smiling (*P* < 0.001). Opioid users surveyed were significantly more likely to feel uncomfortable in the presence of close relatives and less likely to participate in social activities (*P* < 0.001). 37% of the OAP reported frequent restless sleep and 12% frequent difficulties in performing daily tasks. No significant differences were found between the test and control groups in speaking or pronouncing words (*P* = 0.054), and opioid users did not report more absences from work (*P* = 0.242).

**Table 9:** Results of the questionnaire about the function of the teeth/chewing organ. Data are expressed as *n* (%). Abbreviations: opioid addicted patients (OAP), non-opioid addicted patients (non-OAP).

	Answer	OAP	non-OAP	*P*-value
Difficulty biting into food	Often	16 (32)	0 (0)	<0.001
	Not often	34 (68)	50 (100)	
Difficulties with chewing	Often	14 (28)	0 (0)	<0.001
	Not often	36 (72)	50 (100)	
Difficulty speaking/pronouncing words^a^	Often	4 (8)	0 (0)	0.054
	Not often	44 (92)	50 (100)	
Dry mouth	Often	19 (38)	0 (0)	<0.001
	Not often	31 (62)	50 (100)	
Feeling embarrassed in public because of the condition of your teeth	Often	22 (44)	0 (0)	<0.001
	Not often	28 (56)	50 (100)	
Tense feeling due to problems with teeth and mouth^a^	Often	21 (43)	0 (0)	<0.001
	Not often	28 (57)	50 (100)	
Avoiding smiling because of your teeth	Often	20 (40)	0 (0)	<0.001
	Not often	30 (60)	50 (100)	
Restless sleep^a^	Often	18 (37)	0 (0)	<0.001
	Not often	31 (63)	50 (100)	
Taken time off work^a^	Often	2 (4)	0 (0)	0.242
	Not often	47 (96)	50 (100)	
Difficulties in carrying out everyday activities	Often	6 (12)	0 (0)	0.027
	Not often	44 (88)	50 (100)	
Reduced well-being, when being with the spouse or other close persons^a^	Often	17 (35)	0 (0)	<0.001
	Not often	32 (65)	50 (100)	
Reduced participation in social activities	Often	14 (28)	0 (0)	<0.001
	Not often	36 (72)	50 (100)	

^a^ Missing answers in the OAP group and therefore only 48 or 49 valid values.

### 3.6 Dietary habits questionnaire

The dietary habits of OAP and non-OAP are shown in Table 10. No significant differences were found in the frequency of consumption of fresh fruit (*P* = 0.412), biscuits, cakes and pies (*P* = 1.0), pastries and buns (*P* = 0.55), jam and honey (*P* = 0.27) or chewing gum with sugar (*P* = 1.0). However, opioid users reported consuming sweets (*P* = 0.027) and drinking lemonade, cola or other sweet drinks such as sweetened tea or coffee (*P* < 0.001) more often than participants in the control group.

**Table 10:** Results of the questionnaire about dietary habits. Data are expressed as *n* (%). Abbreviations: opioid addicted patients (OAP), non-opioid addicted patients (non-OAP).

	Answer	OAP	non-OAP	*P*-value
Fresh fruit	Often	28 (56)	33 (66)	0.412
	Not often	22 (44)	17 (34)	
Biscuits, cake, pie	Often	20 (40)	19 (38)	1.0
	Not often	30 (60)	31 (62)	
Pastries, bread rolls^a^	Often	29 (58)	25 (51)	0.55
	Not often	21 (42)	24 (49)	
Jam or honey^a^	Often	16 (33)	11 (22)	0.27
	Not often	33 (67)	39 (78)	
Sugary chewing gum	Often	4 (8)	4 (8)	1.0
	Not often	46 (92)	46 (92)	
Sweets^a^	Often	30 (61)	19 (38)	0.027
	Not often	19 (39)	31 (62)	
Lemonade, cola or other sweet drinks	Often	30 (60)	7 (14)	<0.001
	Not often	20 (40)	43 (86)	
Sweetened tea	Often	27 (54)	8 (16)	<0.001
	Not often	23 (46)	42 (84)	
Sweetened coffee	Often	32 (64)	10 (20)	<0.001
	Not often	18 (36)	40 (80)	

^a^ One missing answer in the OAP group and therefore only 49 valid values.

## 4 Discussion

Despite the growing threat of opioid addiction and its significant health consequences, few studies have focused on the oral health of drug users, particularly opioid addicts (23, 24, 29). Existing research is often limited to specific populations, such as women or users of specific substitution drugs, and is always influenced by the particular characteristics of the site or region where it is conducted (23, 29, 30). In particular, there is a lack of evidence on the oral health of opioid-dependent patients in central European countries, especially in Germany. This retrospective cohort study fills a gap by examining the oral health, dental hygiene, dietary habits, self-perceived pain, and functional limitations in a cohort of opioid users. The results show differences between opioid-dependent and non-opioid-dependent patients in these categories, justifying rejection of the null hypothesis.

A major strength of this study is the inclusion of a matched non-OAP cohort, which allows objective comparisons to be made on all the parameters studied. Age and sex matching between the OAP and control groups increases the validity of the study in assessing oral health outcomes. However, some confounding variables, such as socio-economic background, were not fully accounted for. Significant differences were observed between the OAP and control groups in terms of education and place of residence. The lower level of education among OAP participants is consistent with German data showing that drug users typically have a lower level of education than the general population (31). In addition, the predominance of urban residence among OAP participants is consistent with Lenardson et al. (32), who reported a higher prevalence of opioid use in cities. However, contrasting reports from the USA suggest that non-medical opioid misuse also occurs in rural areas (33, 34). In this study, the urban residence of OAP participants probably reflects two factors: (i) easier access to drugs in cities in eastern Bavaria compared to rural areas, and (ii) the requirement that substitution treatment take place in an urban clinic (Department of Psychiatry and Psychotherapy, University of Regensburg), where the study cohort was recruited. Another potential confounding factor concerns the control group recruited from the Department of Conservative Dentistry and Periodontology. These individuals received regular high-quality oral health assessments, which may introduce selection bias. Control patients may inherently have better oral health and dental hygiene than the general population, potentially exaggerating differences between OAP and controls. The regular exposure of controls to professional dental care contrasts sharply with the likely limited engagement of OAP.

The main drug of abuse in the cohort of OAP was heroin, closely followed by cannabinoids and cocaine. Although opioid use was an inclusion criterion in this study, other studies have also reported heroin as the second or even the most commonly used drug (23, 35–37).

Gateway drugs used at an early age were sniffing substances, alcohol and cannabinoids. However, alcohol was also a frequent companion throughout the addiction phase and was used over a long period of time. In a Spanish cohort of drug users, half were chronic alcoholics, similar to the findings of this study (37).

The included cohort of OAP was recruited from substitution therapy and received appropriate medication and close medical care. In general, methadone is described as the most commonly used medication in substitution programs, followed by buprenorphine, levo-alpha-acetylmethadone (LAAM) and dihydrocodeine (35, 38–41). However, at the Centre for Addiction Medicine of the Department of Psychiatry and Psychotherapy at the University of Regensburg, patients were mainly treated with Polamidon (LAAM) or Subutex (buprenorphine). The reason for this is that the substitution regime is individually tailored to each patient in a “shared decision”-approach with the treating physicians. The main objective is to achieve safe and sustainable withdrawal from illicit opioid use. In order to optimize compliance and feasibility for patients, their individual previous experiences and wishes are integrated into the substitution plan, thereby also reducing side effects. If previous drug experience is still close, (D-) L-methadone is usually chosen, as many patients subjectively feel that it has a better shielding effect. L-methadone is usually preferred because of its lower rate of side effects. In the advanced stages of substitution treatment, buprenorphine is often used because patients report that they have better cognitive awareness and therefore fewer problems in everyday life or at work.

Significant differences were found in the oral health status of OAP and non-OAP. In terms of dental status, the median DMFT of opioid-dependent participants was significantly higher due to a large number of decayed and missing teeth. This means that the DMFT is even twice as high as that of the German cross-section of young adults (42). The DMFT of the control group, however, corresponds to the German average as determined in the German Oral Health Study V (42), which shows that the control cohort was representative and therefore effective. A similarly high DMFT, as described in this study, has also been reported in numerous other studies investigating opioid-addicted patients (23, 37, 43).

The assessment of periodontal health in this study revealed a significantly higher need for periodontal treatment in the test group. Status was assessed using the PSI, an index widely used by German dentists to define the periodontal treatment need. Existing evidence is often derived from the Community Periodontal Index of Treatment Need (CPITN), from which the PSI was derived, making both indices comparable (26). In general, other studies have also observed a high prevalence of periodontitis in opioid users (23, 44, 45). By contrast, Ma et al. (39) reported a lower rate of periodontal pockets, but only 10 index teeth were assessed and the number of missing teeth, possibly for periodontal reasons, was much higher.

Plaque accumulation on the tooth surface, an indicator of oral hygiene, was measured using the PI according to Silness and Loe. OAP were significantly more likely to have a PI categorized as ‘poor’ than participants in the non-OAP group. Similarly poor oral hygiene was reported for heroin users by Mehmood et al. (46). A possible explanation for the poor oral health can be found in the lifestyle of addicts. Studies have shown that opioid addicts have a preference for sweet flavors and meals high in sugar, both increasing risk of caries (47, 48).

Regarding the investigated saliva parameters of OAP, the literature presents controversial findings. While some authors, such as Protrka et al. (43), have reported a reduced flow rate of both unstimulated and stimulated saliva in drug users, suspecting that it could be due to smoking or taking medication or drugs that lead to xerostomia, the present study did not find significant differences in stimulated salivary flow rate or buffer capacity. Del Ribeiro et al. (49) recorded a mean salivary flow rate of 1.31 ml/min, which is within the physiological range and comparable to the flow rate observed in both OAP and non-OAP. The saliva has a bicarbonate buffer system that counteracts acidic cariogenic attacks. As saliva secretion increases, its bicarbonate content rises, resulting in a higher pH. This neutralizes the organic acids in plaque and reduces the risk of caries. Also, higher salivary flow rate yields a higher oral clearance, supporting increased pH-values (50). Conversely, low salivary flow is associated with limited buffering capacity. Consistent with the findings for OAP in this study, Mateos-Moreno et al. (37) measured a mean saliva pH of 6.8 in drug users, which was significantly different from the non-OAP group. The acidic salivary pH in OAP may be due to poor oral hygiene and high cariogenic activity. Setiawan et al. (51) showed that salivary pH increases after tooth brushing, suggesting that poor oral hygiene is a trigger for acidic salivary pH and high cariogenic activity.

The questionnaire aimed to analyze oral hygiene behavior and dietary habits, both of which may indirectly affect the oral health of drug users. It also included a self-assessment of dental health and quality of life.

OAP appeared to brush their teeth significantly less often, as several studies have shown (36, 45, 52). However, it was not just the frequency of brushing that was reduced, the quality was also inferior. The OAP in the present study showed significantly poorer interdental hygiene, with a lack of use of dental floss and interdental brushes, a finding confirmed by other studies (36, 52). In addition, awareness and knowledge of oral hygiene among drug users appeared to be limited. For example, 46% of drug users surveyed did not know whether their toothpaste contained fluoride, an important factor in caries prevention, compared to 28% in the control group (53).

Significant differences in dietary habits were found between the test and control groups. Although no increase in the consumption of traditional sugary foods like fruit, cakes, or pastries was observed, OAPs consumed significantly more sweets and sugary drinks—a trend consistently reported in numerous studies (36, 52, 54). This may be due to physiological and hormonal changes during drug withdrawal that affect food intake, nutrient absorption and the regulation of hormones linked to appetite and satiety (47). Furthermore, people addicted to opioids often replace protein and fat with sugary meals due to an increased preference for sweet tastes (55). Especially during substitution treatment, cravings for sugary foods, such as cakes and sweets, are often increased as a substitute for the drug (47). In addition, socio-economic factors, as indicated by lower levels of education in the test group, may also play a role by limiting access to a healthy, balanced diet. These findings highlight the need to promote a healthy, low-sugar diet for OAPs, not only to reduce the risk of sugar-related oral diseases such as dental caries, but also to improve overall health and well-being (56).

Drug users rated their dental status as significantly worse than patients in the control cohort, which largely corresponded to the objectifiable results of the dental examination. Although the OAP were well aware of their poor oral health and the limitations it imposed on their daily lives, they rarely visited a dentist. More interestingly, a clinical trial in Lithuania reported lower DMFT in OAP who visited the dentist at least once a year, highlighting the importance of prophylaxis and dental check-ups to maintain dental and periodontal health (30). Despite this, only a fraction of the OAP in this study underwent regular routine check-ups. 56% of OAP consulted the dentist only because of pain or other discomfort, which could have been avoided with regular prophylactic measures.

A reduction in the quality of life of opioid users was evident in many ways, including difficulties with eating, dry mouth and associated social problems, as shown by Mehmood et al. (46). For many, the poor condition of their teeth led to shame and embarrassment, resulting in reduced social interaction. The OAPs also reported a limitation to perform daily activities, however, no increase in absenteeism was found compared to the control group. This could be due to factors such as a heavy dependence on income from work, occupational structure or potential unemployment. However, the available data do not allow any definitive conclusions to be drawn.

A key strength of this retrospective cohort study is the inclusion of a matched control group, allowing objective comparison across multiple oral health dimensions. The comprehensive assessment of dental, periodontal and salivary parameters, together with self-reported behaviors and functional limitations, provides a holistic understanding of the oral health challenges faced by OAP. However, limitations include potential selection bias due to the recruitment of OAP exclusively from an urban substitution treatment center, which limits the generalizability of findings to rural or medically unsupervised populations. In addition, the retrospective nature of the study and reliance on self-reported data for certain measures may introduce recall bias or inaccuracies. Despite these limitations, the study fills an important gap in understanding the oral health of opioid users in Germany.

The clinical implications of this study are substantial and multifaceted. The findings highlight the urgent need for targeted oral health interventions for OAP. The high prevalence of dental decay, periodontal disease, and poor oral hygiene behaviors underscores the importance of integrating dental care into addiction treatment programs. Prioritizing routine dental check-ups, preventive care, and education on proper oral hygiene and dietary habits is essential to address these disparities. In addition, promoting caries prevention strategies, such as fluoride use and reduced sugar intake, should be integral to comprehensive care.

## 5 Conclusion

Opioid addiction is a growing problem in Germany, with a particular impact on oral health. Especially poor oral hygiene and dietary habits lead to common dental and periodontal diseases among opioid addicted patients, resulting in pain, functional limitations and tooth loss, which significantly affect their quality of life and social behavior. Social exclusion and feelings of shame, especially in public places such as a dental waiting room, are often underestimated factors affecting the dental care of drug users. Therefore, it is essential to establish easily accessible, confidential treatment services tailored to the needs of this vulnerable population. Good oral health is essential not only for the daily lives of those affected, but also for the rehabilitation process of drug users, as it has a profound impact on self-esteem and social integration.

## Data Availability

The original contributions presented in the study are included in the article, further inquiries can be directed to the corresponding author.
